# Gender Differences in Witnessing and the Prevalence of Intimate Partner Violence from the Perspective of Children in Finland

**DOI:** 10.3390/ijerph18094724

**Published:** 2021-04-28

**Authors:** Johanna Hietamäki, Marjukka Huttunen, Marita Husso

**Affiliations:** 1 Special Services, Finnish Institute for Health and Welfare (THL), 00271 Helsinki, Finland; johanna.hietamaki@thl.fi; 2 Shelter Mona, Monika-Naiset Liitto ry, 00580 Helsinki, Finland; marjukka.huttunen@monikanaiset.fi; 3 Faculty of Social Sciences (SOC), Tampere University, 33014 Tampere, Finland

**Keywords:** intimate partner violence, children witnessing violence, violence against parents, exposure to violence, child victim survey

## Abstract

Background—Intimate partner violence (IPV) has both direct and longer-term effects on children’s well-being. Much of the research thus far has relied on caregiver reports of IPV and clinical samples of children. By contrast, minimal research has examined violence between parents from the perspective of children using nationwide samples. Objective—This study explored the frequency of IPV witnessed by children and gender variations regarding the victims, perpetrators, and witnesses. Methods—The data were derived from a sample of 11,364 children from the Finnish Child Victim Survey 2013. The children were between 11 and 17 years old and were enrolled in the Finnish school system. The main methods of analysis included crosstabulation and the chi-square test. Results—The results indicate that children witnessed more IPV against their mother (4.9%) than their father (3.5%). Girls reported having witnessed more violence against both their mother (7.0%) and father (5.1%) than boys did (mothers 2.7%, fathers 1.8%). Girls’ reports of IPV against both parents were twice or more than twice as common as boys’ reports. Conclusions—The above differences might result from gendered expectations and boys’ and girls’ different relationships to violence, as well as differences in the recognition and interpretation of violent incidents. Therefore, practitioners should adopt a gender-sensitive approach as a precondition and practice for working with children in social and health care.

## 1. Introduction

This article focuses on the frequency of violence between parents from the perspective of children aged 11–17 years. Intimate partner violence (IPV), which reflects gendered and imbalanced power relations in close relationships, is deeply embedded in societies. Statistics indicate that the rate of IPV in Finland is high compared with other Nordic and European countries [1]. According to Finnish homicide statistics, 59% of adult female victims of homicide were killed by an intimate partner between 2010 and 2018 [2]. Globally, as many as 30% of women have experienced physical or sexual IPV at some point in their lives [3]. IPV has a significant impact on well-being. In addition to human suffering and mental and physical illnesses, violence leads to significant financial costs for the police, the judicial system, and the healthcare and social welfare service systems [1,4].

Few studies have employed nationwide samples to investigate violence between parents from the perspective of children [5,6,7]. Much of the research thus far has relied on caregiver reports of IPV and clinical samples of children, for example, from shelters [7,8,9,10]. Often, these samples have also been small [9,11] and collected from adults [12,13]. In addition, many studies have measured only violence against the mother but not the father [9]. Importantly, various studies have highlighted that violence has both direct and longer-term effects on children’s well-being [8,14,15,16,17,18,19] and affects their parenting [20] and IPV perpetration in adulthood [21].

In this article, we explore the frequency of IPV that children witness and gender variations in victims, perpetrators, and witnesses. In the following sections, we first define the main concepts that appear in the article and then provide a brief review of the literature concerning children’s exposure to violence at home and gendered violence in general. We then present the data and methods of the study. Our data consist of answers to the nationwide (*N* = 11,364) Child Victim Survey. Using these data, we analyzed how common violence between parents was in the reports of children and the gender distribution. The analysis consistently focused on gender as a perspective that operates within various settings and contexts. That is, our premise was based on the performative nature of gender and its complex intertwinement with violence and violent behavior. To conclude, we discuss the reasons for and meanings of the gendered nature of violence and propose questions for further research.

## 2. Literature Review

### 2.1. Children as Witnesses of Intimate Partner Violence

The adoption of intimate partner violence (IPV) as the concept in this study represents both the form of violence that we chose to examine and the content of the violence. With children, we primarily use the concept of witnessing violence. Furthermore, the concept of exposure to violence is common in international research. The concept of experience is particularly common in child-oriented research that considers children as agents and subjects. [7,15,22,23]. The emphasis on the notion of experiencing violence is on the holistic nature of the experience of violence and the fact that the consequences of seeing or hearing violence are not limited to the moment of witnessing the situation, as the child can also witness the effects of violence and be made aware of it otherwise [8]. This perspective is highly important and well founded. However, in the context of our study, we decided to discuss children witnessing violence because it would allow us to present results more clearly.

Estimates of the number of children exposed to IPV between their parents vary significantly in Finland, as well as internationally, and they are rarely based on children’s own reports [5,7,13]. According to Finkelhor et al. [6], 5.8% of children had witnessed IPV during the past year and 15.8% during their lifetime. Furthermore, 7.7% of girls and 4.2% of boys had witnessed IPV within the past year [6]. In Finland, a 2008 survey showed that 11% of children had witnessed violence toward at least one parent during the previous year [24]. It is important to note that children do not necessarily have to witness violence as it happens to notice its existence [25]. Thus, our results do not directly represent the amount of IPV but observations of violence that children have made and reported.

Witnessing IPV at home can influence children’s psychological, emotional, and cognitive activities in various ways. Children with experiences of violence, for example, have more psychological problems, behavioral disorders, and problems at school, and they exhibit above-average violent behavior, especially toward parents [7,26,27,28,29,30]. Although several studies have found support for the hypothesis of intergenerational transmission of violence, some of the results have been contradictory or have shown no difference between genders in intergenerational transmission [7,16,21,31]. Furthermore, children’s exposure to violence is probable if violence has occurred between the parents [7,27,32].

Especially in Scandinavian research, problems relating to the interpretation and meaning-making of parents’ violent behavior have been quite prominent in child-oriented research on IPV [33]. Findings indicate that children learn at an early age to regard violence as a negative issue but also to associate it with manliness and masculinity. In children who witness their father commit violence against their mother, this can provoke an internal conflict about being able to regard the father as a role model or loving parent but still viewing violence negatively [15]. Moreover, children’s emotions toward their parents can be quite ambivalent if they have to balance between loyalty and turning against a parent perpetrating violence, or between compassion and protectiveness, as opposed to guilt and hatred toward the mother as a victim [14,15,28].

Research shows that violence is gendered in multiple ways, and the effects of violence are also deeply intertwined with gender for children. For instance, it seems that this likely manifests in boys as external hostility and aggressiveness, whereas girls are likely to exhibit internalized difficulties, such as depression and somatic complaints [5,17]. The gender of the abusing parent, as well as the victim, can also affect how children react to violence, and girls and boys often have different means of coping after exposure to abuse [34]. However, the studies on how children encounter and experience violence have usually only stated the existence of gender differences or similarities. Thus, the links between violence and gender, as well as related effects on children’s conceptions and activities, remain a largely unexplored research area.

### 2.2. Gendered Violence as a Perspective

IPV research acknowledging the significance of gender originated in the Anglo-American research tradition of the 1960s and 1970s. Considerable statistical research on violence against women was necessary to make a gender-sensitive approach to research mainstream [35]. It was only in the 1990s, when the first large-scale statistical study on violence against women in Finland revealed the extent of the phenomenon, that Finnish IPV researchers began paying attention to the gendered nature of this phenomenon [36]. According to more recent statistics, almost one-third of women in Finland have experienced physical or sexual violence committed by their present or previous partner. With ex-partners, the number is even higher: half of all women have experienced violence or threats at the hand of a former male partner [1,37]. Even though the topic was quite visible throughout the 2000s, there was no significant change in the amount of violence during this time [37,38,39]. Internationally, the proportion of women who have experienced physical or sexual violence in intimate relationships varies from 15 to 71%, with a global average prevalence of 30% [3,40].

Men also face violence in intimate relationships, although there are both qualitative and quantitative differences between genders. For instance, women more often endure repeated violence and suffer injuries from this violence. IPV causes women physical injuries more than twice as often and mental consequences more than three times as often compared to men. Women’s injuries are also more severe, and women experience the threat of violence as mentally harmful more often than men do [41].

Previous studies have used, for example, biological and psychological explanation models to theoretically explain IPV [42]. The present study relied on the discourses of social sciences and gender studies, which treat gender as a segregation tool that is produced through structures, practices, activities, behaviors, and lived relationships. We consider gender as a combination of performance and gendered habits and behaviors—that is, one performs and repeats the gestures and conduct associated with femininity and masculinity [43,44]. Accordingly, gender formation is a lifelong, interactive process [45,46]. Violation, violent representations, practices and ideologies, and violent behavior also influence gender, but the common interpretation is that violence is gender-based rather than something that constructs and creates gender [47,48,49].

Although gender does not explain violence or nonviolence, violence and the threat of it also essentially shape the relationship between genders. We live in a culture where violence is perceived as a symbol of masculinity and masculine corporality is created and valued through displays of power and force [50,51]. For instance, some types of violence are acceptable and even honorable in our society, such as the official monopoly on violence represented by the police and the army, whereas other types of violence are defined as criminal acts and sanctioned. However, a common feature of these forms is that they are mainly controlled by men and closely linked to the performance of masculinity [52,53]. Often underpinning such masculinity are force, power, and competition, and the ability to use violence as a tool to solve problems is part of this process [50,54].

## 3. Purpose, Data, and Method

The aim of the present study was to contribute to the knowledge of IPV from the perspective of children who have witnessed it in their families. Therefore, the focus was on the frequency of IPV against parents as reported by children. A particular topic of interest was how IPV and witnessing it were distributed according to gender. First, using children’s survey responses, we mapped differences between the shares of mothers and fathers as perpetrators and victims of violence. Second, we examined differences between girls and boys in how they reported IPV against their parents.

The study was based on data from the Finnish Child Victim Survey that was conducted in 2013 by the Police University College [55]. The sampling was made as a stratified cluster sample based on county, quality of the municipality, and size of the school [56] (p. 177). The original sample size was 21,825 pupils, 11,364 of whom completed the survey. They were in years six (11–13 years old) and nine (14–16 years old) of the Finnish education system. Pupils in year six accounted for slightly more than half (55.2%) of the respondents. Girls represented 50.4% (*n* = 5731) of the sample, boys represented 49.2% (*n* = 5592) of the sample, and 0.4% (*n* = 44) did not specify their gender. There was no systematic loss of data. The pupils answered the web-based, structured survey during a lesson in school. The survey mapped the respondents’ life situations and experiences of crime, violence, and bullying [55].

The survey covered a wide variety of questions related to the different forms of violence against children at home, school, street violence, and sexual abuse. For the present study, we selected answers to questions concerning violence against parents from the aforementioned data. The examined variables concerned IPV against mothers or fathers. The Violence against Parents measure was developed by the Norwegian Social Research Institute [57]. Violence against the mother was measured with the following question: “Have you seen or heard any of the following happening to your mother in your home in the past 12 months?” The survey included similar separate questions related to violence against the father. The acts of violence were categorized as follows: “She has been called names,” “She has been mocked or disparaged,” “She has been threatened with violence,” “She has been pushed or shaken violently,” “Her hair has been pulled,” “She has been slapped,” “She has been hit with a fist,” “She has been hit with an object,” “She has been beaten up,” “She has been attacked with a knife,” “She has been threatened with a gun,” and “She has been a victim of some other violent act.” The response options were “No” and “Yes.” If any answer to the question about acts of violence was “Yes,” there was a follow-up question about the perpetrator: “Who was the person who did the things mentioned above to your mother? You can select more than one option.” The response options were “Father,” “Stepfather,” “Brother,” “Sister,” “Myself,” “Another relative (who?),” “Another person (who?).” For our analysis, we selected cases where the mother had been the target of the father’s or stepfather’s violence and cases where the father had been the target of the mother or stepmother.

We employed IBM SPSS Statistics 25.0 (SPSS Finland Oy, Espoo, Finland) to analyze the data and we used descriptive data analysis (frequencies and percentages), crosstabulation, and the χ² test to examine differences between groups (gender of children and parents). As the variables concerning the form of violence and the perpetrator were separate, they were combined into new variables. This highlighted which children had expressly witnessed violence between their parents. First, we formed a variable of IPV by itemizing cases in which the respondent had answered “Yes” to any form of violence (see Table 1). This enabled us to determine whether the child had witnessed IPV. Second, we joined the 12 forms of violence against parents into compound variables constituting three categories: (a) psychological violence, (b) mild physical violence, and (c) severe physical violence (see Table 3). Our categorization was based on the difference between petty and aggravated assault in the Criminal Code of Finland [58]: in aggravated assault, the perpetrator uses a firearm, edged weapon, or another comparable instrument, except for the slightly vague concept of “beating up,” which also represents aggravated assault.

We created variables representing accumulation—that is, the occurrence of several forms of violence—by regarding the response options such that each of the three forms of violence witnessed (psychological, mild physical, and severe physical) were added together. These variables then made it possible to create a variable describing the accumulation of IPV, which encompassed at least one and a maximum of three forms of violence.

The study was prepared and conducted according to the Finnish research ethics guidelines in social research, which are administered by the Finnish Advisory Board on Research Integrity. The data collection followed guidelines for anonymous social research conducted on children in Finland [59]. Furthermore, the children’s right to express their will in all decisions concerning them is based on the United Nations Convention on the Rights of the Child (1989). The children were the key persons when it came to deciding whether to participate in the survey. Participation in the survey was voluntary and this information was given to the children. The researchers who collected the data were well aware that voluntary participation can be seen as a complex issue in any institutional setting for children. “The heads of the schools had the authority to decide if the research would be carried out in their schools. The children’s parents were informed that a study of this nature had been conducted at school after the study had been completed” [60].

It should be noted that we can only know the number of reported cases but not the actual observations, which is why we speak about reporting of witnessing in this article. In addition, due to the breadth of the topic, this study could not take into account the relationship between violence against children and violence between parents. Furthermore, if a parent had faced violence not only from the partner but also from children or other relatives, the data did not specify which forms of violence were traceable to the partner and which ones were traceable to someone else. This is also a limitation of the analysis method. There were 102 cases in which the father or stepfather and some other family member had perpetrated violence against the mother. In total, 526 children had witnessed IPV against the mother. Therefore, in about one-fifth of all IPV cases against mothers, the mother had faced other sources of violence as well. Conversely, the mother or stepmother and another family member had committed violence against the father in 88 cases, equaling about one-fourth of the IPV cases against fathers, totaling 367 child witnesses.

## 4. Results

### 4.1. Intimate Partner Violence Witnessed by Children

The frequencies and percentages of IPV against the mother and the father that boys and girls reported appear in Table 1. The results indicate that slightly more than 6.3% of the children reported having witnessed IPV against one of their parents at home during the past year. There were clear gender differences in the children’s IPV reports. Respondents reported having witnessed IPV against mothers more frequently than against fathers. Another gender difference was that girls reported having witnessed IPV against both their mother and father more often than boys.

As shown in Table 2 below, 2.1% of respondents reported having witnessed IPV against both parents. In observations of IPV against only one parent, the mother was the target more often than the father. Nearly 94% of respondents reported not having seen or heard IPV against either of their parents.

### 4.2. Intimate Partner Violence by Form and Parent Gender

**Figure 1:**
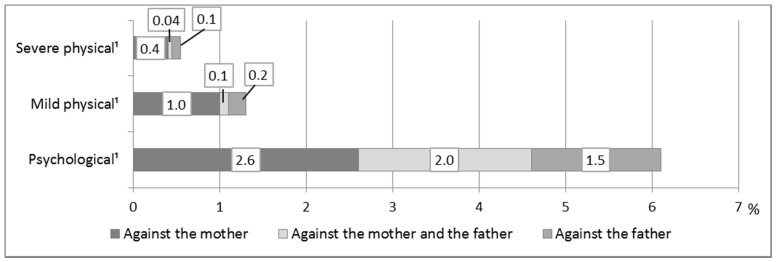
Forms of intimate partner violence against mothers, both parents, and fathers (*N* = 10,356–10,451). Note. ¹ Exact Test (*p* < 0.001).

According to the results, psychological violence was the most common form of IPV (Table 3). More children reported having witnessed fathers perpetrating psychological violence against mothers than the other way around. According to their answers, name-calling was the most common form of IPV against both mothers and fathers. Mocking and disparaging were also more common than mild physical violence. There were very few reports of fathers being the targets of other forms of IPV. The data also show differences between mothers and fathers regarding whether both parents or only one was the target of psychological violence. Typically, only the mother experienced psychological IPV. The second most typical case was that violence was reciprocal, and the least typical case was that only the father experienced psychological IPV (Figure 1).

The gender difference between the parents seems to be even clearer when it came to mild physical violence. Four times more children reported having witnessed violence against mothers than against fathers (Table 3). In addition, mild physical violence in the family exclusively against the mother was clearly a more typical case than violence exclusively against the father (Figure 1). The most severe forms of physical violence, such as hitting with an object, attacking with a knife, and threatening with a gun, were the least common incidents. Twice as many respondents reported having witnessed severe physical violence against mothers than against fathers (Table 3). They also reported mothers as typically being the exclusive targets of this violence. The severe forms of physical violence were less commonly reciprocal or targeted exclusively at fathers (Figure 1).

### 4.3. Differences between Girls and Boys

**Figure 2:**
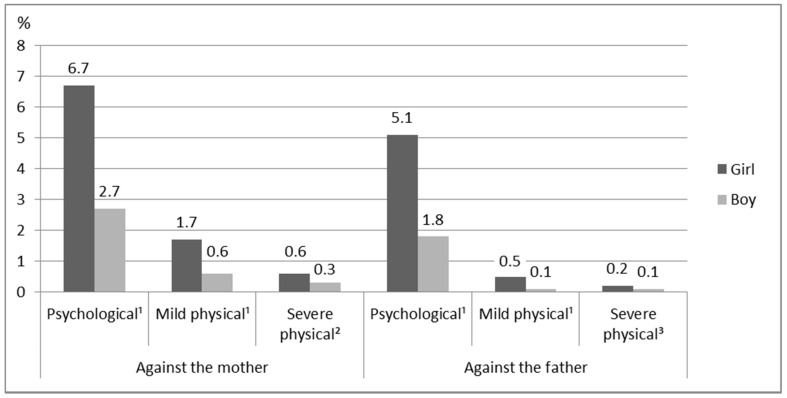
Percentages of intimate partner violence against mothers and fathers as witnessed by girls and boys (*N* = 10,505–10,603). Note. ¹ Exact Test (*p* < 0.001). ² Exact Test (*p* = 0.013). ^3^ Exact Test (*p* = 0.147).

The results indicate that there was a clear difference between girls and boys in terms of reports of having witnessed IPV against one or both of their parents. The girls’ answers indicate that they had seen or heard all forms of IPV more often than boys had, regardless of whether the target of the violence was the mother or father (Figure 2). Girls reported psychological and mild and severe physical IPV against both parents more than twice as frequently as boys did.

**Figure 3:**
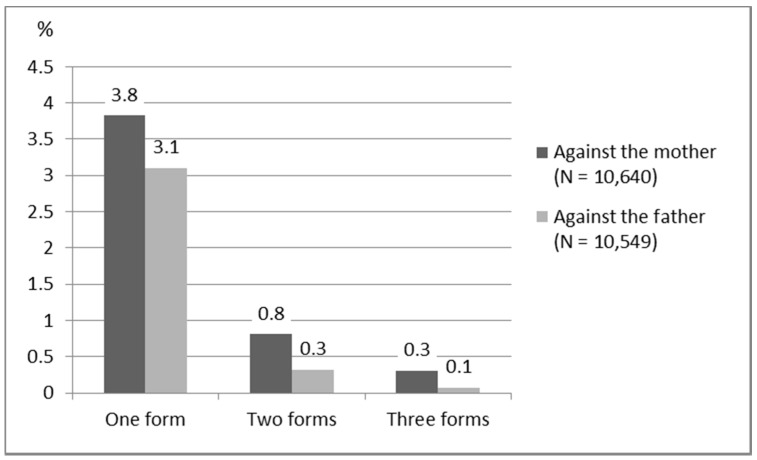
Percentages of forms of intimate partner violence (psychological, mild physical, and severe physical) against mothers and fathers.

In situations where different forms of violence occurred simultaneously, the results show a common co-occurrence of psychological violence. If the mother endured physical violence, 85.4% (*n* = 137) of such cases also included psychological violence, and when the father was the target of physical violence, 95.5% (*n* = 44) of such cases included psychological violence. The most common case was children reportedly having witnessed one form of IPV against a parent, which was usually psychological violence (Figure 3). The three forms of violence seldom occurred simultaneously. Mothers were the target of accumulated violence more often than fathers. Mothers were also more likely than fathers to face one to three forms of violence (*p* < 0.001).

**Figure 4:**
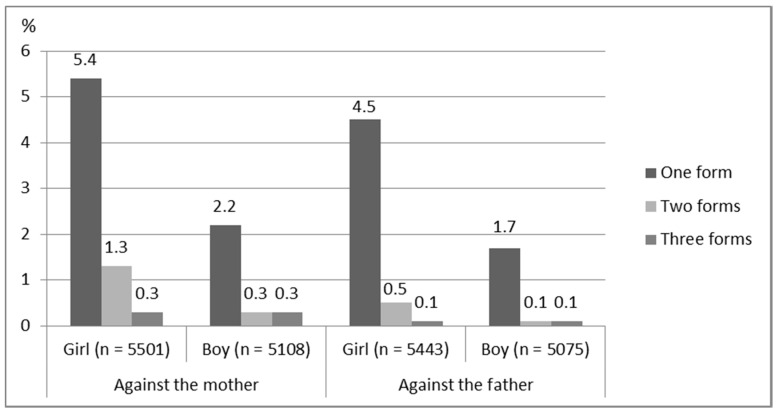
Percentages of forms of violence against mothers and fathers witnessed by girls and boys.

In light of these results, it seems that girls reported having witnessed one to three forms of IPV against mothers more than boys did (exact *p* < 0.001; Figure 4). Both genders most commonly witnessed one form of IPV. The number of reports of one or two different forms of violence was more than twice as high for girls as it was for boys.

A difference between girls and boys was also visible in their answers relating to violence against fathers (exact *p* < 0.001; Figure 4). The results indicate that both girls and boys very seldom witnessed several simultaneous forms of IPV against fathers (less than 1%). Among those who reported having witnessed one or two forms of IPV against fathers, the number was twice as high for girls as it was for boys.

## 5. Discussion

According to the results, 6.3% of children reported having witnessed IPV against one of their parents during the past year. Curiously, this figure is significantly lower than the corresponding frequency of 11% found in Huttunen et al.’s [24] study, which was based on the same kind of data from the Finnish Child Victim Survey conducted in 2008. According to the most recent Finnish studies on crime victims, the number of violent crimes and cases of IPV have remained stable in recent years [38,39]. Hence, the statistics available do not show such drastic changes in the frequency of IPV [1,37,38]. Nevertheless, the change in children’s reports of having witnessed IPV provokes questions for further research, such as whether IPV against parents has decreased, whether it has become more difficult for children to report their experiences of IPV, or whether IPV has become normalized and difficult to recognize or name as violence in a way that prevents children from reporting and disclosing certain acts as violence.

Among the acts of IPV that this study investigated, the most common were name-calling, mocking, and insulting, which we defined as psychological violence. These acts of violence were not included in previous Finnish victim surveys [37]. Therefore, there is very little prior research data available on their frequency. Nevertheless, we should not underestimate the significance of name-calling and mocking as degrading forms of violent behavior. The power dynamics within the family can influence children’s ideas about gender relations and related power structures [61,62]. Parents’ actions in their close relationships and intergender interactions thus have a major influence on children’s perceptions of gender norms and gendered behaviors.

According to our results, children reported having witnessed psychological violence more often than physical violence, and it seems that mothers clearly endured it more often than fathers did. When we examined the accumulation of IPV—that is, the occurrence of several forms of violence in the same family—the results indicated a similar gender difference regarding the other forms of violence. Women had faced one to three forms of violence more often than men. In light of earlier research on the dynamics of IPV, it is not surprising that psychological violence against women often escalates to physical violence [63]. In our culture in particular, the use of physical violence is more acceptable from men than it is from women [43,50,52].

In conclusion, the results suggest that IPV is gendered at two levels: there seem to be clear gender differences in the reported frequencies of IPV against mothers and fathers and of girls and boys having witnessed IPV against mothers and fathers. In the children’s reports, witnessing IPV against mothers was more common than against fathers. There was an even clearer gender difference regarding respondents who had witnessed violence: girls reported having witnessed all forms of IPV against both parents much more often than boys did. There could be at least two reasons for this difference. Either the difference is real, in the sense that boys really do witness fewer incidents of IPV than girls do, or girls and boys interpret violence and report their observations of violence differently.

## 6. Conclusions

Based on our theoretical framework, we suggest that the dissimilarities in the reported frequencies of IPV against mothers and fathers and of girls and boys having witnessed IPV against mothers and fathers may result from gender differences in how boys and girls recognize, encounter, and interpret violence. In our culture, girls and boys have different relationships to both the use and experience of violence [64,65]. This might affect, for instance, what they interpret as violence, how they experience and explain violence, and how they believe they should respond to violence.

A gendered relationship to violence, as well as gendered expectations and assumptions regarding behavior related to violence, is also visible in children’s everyday lives. Scuffling, which we may characterize as a form of physical violence, is still regarded as a normal part of interactions between boys, and girls and boys cultivate different attitudes toward expressions of aggression and anger or the use of violence. For example, in situations where boys perceive violence as a demonstration of control and power, girls easily interpret it as a loss of self-control [65]. Survey participation and response styles in surveys might also be gendered [66]. Research has shown that gendered behavior already appears in the answers of children in years 3–6 of primary school. Boys have been found to avoid revealing their feelings and expressing weakness and vulnerability considerably more than girls do, and boys have also been shown to have a more permissive attitude toward violence as a problem-solving tool [67]. Furthermore, research on the coping mechanisms of girls and boys indicates that gendered behaviors and expectations also affect children’s ways of coping and seeking help. Studies have shown that girls emphasize social relationships more than boys do, and boys deny problems and try to manage by themselves more often than girls do [7,34,68]. However, in cases where fathers have subjected mothers to IPV, both girls and boys have described their father as an irresponsible care provider and their mother as responsible for parental care [69,70].

Many child welfare workers in Finland and other Nordic countries have regarded children exposed to IPV as high-risk cases for abuse and neglect [71]. In many countries, children also have an important role in child protection decision making and interventions in cases of IPV [72]. In addition, there are different kinds of psychosocial, individual, trauma-focused, family-based, and group interventions for children exposed to IPV [73,74]. Hence, children “can no longer be described as forgotten victims, as witnessed by the fact that there is growing recognition of their human rights and a stated intention to listen to what they have to say” [75] (p. 188). However, despite that, there seem to be shortcomings in children’s real participation in decision making regarding child protection and evaluation of the need for violence interventions [76], and only a few working methods for abused children take account of gender differences [77].

Gendered behaviors and expectations, in addition to the gendered nature of coping mechanisms, might partially explain the significant differences in the reported frequencies of IPV against mothers and fathers between girls and boys. Hence, the results suggest that women and men, as well as girls and boys, might have a gendered relationship to violence. This observation has significant consequences for both research and practice. Practitioners working with children in social services, health care, and educational settings should adopt a gender-sensitive approach as a precondition and practice for working with children. Awareness of gender differences in relation to violence is important for future research, as well as welfare and violence-prevention services in supporting victims and witnesses of IPV.

## Figures and Tables

**Table 1:** Intimate partner violence against parents witnessed by girls and boys (*N* = 10,519–10,699).

Gender	Against the Mother ^1^ % (*n*)	Against the Father ^1^ % (*n*)	Against the Mother and/or the Father ^1^ % (*n*)
Girls	7.0 (386)	5.1 (280)	9.1 (501)
Boys	2.7 (139)	1.8 (92)	3.4 (177)
Total	4.9 (525)	3.5 (372)	6.3 (678)

^1^ *p* < 0.001.

**Table 2:** Differences in intimate partner violence against mothers and fathers (*N* = 13,459).

Against the Father	Against the Mother		
	No % (*n*)	Yes % (*n*)	Total % (*n*)
No	93.7 (9806)	2.7 (282)	96.4 (1088)
Yes	1.5 (153)	2.1 (219)	3.6 (372)
Total	95.2 (9959)	4.8 (501)	100.0 (10,460)

*p* < 0.001.

**Table 3:** Frequency of forms and acts of violence against parents (*N* = 10,416–10,611).

Form of Violence	Against the Mother % (*N*)	Against the Father % (*N*)	*p*-Value	Total % (*N*)
Psychological violence	4.7 (505)	3.5 (370)	0.000	6.1 (634)
Name calling	4.3 (459)	3.2 (340)		
Mocking or disparaging	2.2 (234)	1.6 (166)		
Threatening with violence	0.9 (93)	0.2 (22)		
Mild physical violence	1.2 (123)	0.3 (35)	0.000 ^1^	1.3 (132)
Pushing or shaking violently	0.9 (95)	0.2 (26)		
Pulling hair	0.4 (46)	0.1 (8)		
Slapping	0.5 (48)	0.2 (20)		
Punching	0.4 (39)	0.1 (11)		
Severe physical violence	0.4 (47)	0.2 (17)	0.000 ^1^	0.5 (54)
Hitting with an object	0.3 (36)	0.1 (13)		
Beating up	0.2 (24)	<0.1 (4)		
Attacking with a knife	0.1 (15)	0.1 (7)		
Threatening with a gun	0.1 (15)	<0.1 (3)		
Other violent act	0.4 (38)	0.1 (10)		

^1^ Exact test.

## Data Availability

Child Victim Survey 2013 data is available: https://services.fsd.tuni.fi/catalogue/FSD2943?lang=en&study_language=en (accessed on 14 August 2019).
